# Supplementary material for: Evaluating the effect of Chinese control measures on COVID-19 via temporal reproduction number estimation
Source: PLoS One. 2021 Feb 11;16(2):e0246715. doi: 10.1371/journal.pone.0246715 (PMC7877593; doi:10.1371/journal.pone.0246715)
Supplement: Attachment — Submitted filename: Reply.pdf [file pone.0246715.s002.pdf]

Dear Editor,

Thank you very much for processing our manuscript entitled “Chinese Control Efficacy on COVID-19”, and thanks for all the valuable comments and suggestions, which provide the excellent guidance to improve our manuscript. Accordingly, we have largely revised the manuscript. Enclosed please find a detailed response to the referee report. For the sake of convenience, the main modifications are marked in red in the revised manuscript. We believe that the revised manuscript can meet the standard of PLoS ONE.

Reviewer #1: General comments –

• Q1. The manuscript needs editing for spell check, English and grammar.

Reply: We have carefully checked the whole manuscript and corrected grammatical errors and unclear expressions.

• Q2. The control methods deployed in China should also be described considering that these were effective to get the R0 to <1 within a week. It may help other countries to streamline their strategies.

Reply: Thank you very much for this constructive suggestion. In the revised version, we have listed representative control methods deployed by Chinese government. Due to these measures, the effective reproduction number was rapidly reduced, and the spread was effectively suppressed. The added descriptions of control measures are as follows (presented in the Discussion of the revised version).

The huge success of Chinese control measures on COVID-19 resulted from the ambitious and aggressive government-led actions. In order to block transmission and reduce public health hazards, the "five early" measures, namely "early detection, early report, early investigation, early isolation and early treatment", are implemented. *Early detection.*—Rapid detection and diagnosis to promote the timely and effective management of confirmed and suspected cases. *Early report.*—Immediate report to the disease control department about confirmed and suspected cases to start investigation and treatment as soon as possible. *Early investigation.*—Quick epidemiological investigation on the exposure and detailed contacts of confirmed and suspected cases. Through such investigation, we can find out the transmission chain of each case, so as to comprehensively manage all possible infected individuals related to each case. *Early isolation.*—All confirmed and suspected cases, as well as their close contacts will be isolated as soon as possible. *Early treatment.*—Quick providing of proper treatment (symptomatic treatment, supportive treatment, antiviral treatment via traditional Chinese medicine, etc.) to prevent the development of symptom.

To efficiently and effectively implement the "five early" measures, some advanced information techniques are employed. For example, in many cities, the QR codes (similar to these used for online payments) are posted in public transport means (buses, subway stations, taxies, etc.), places with possible crowds (supermarkets, bazaars, restaurants, office buildings, etc.) and places worth particular attention (drugstores). People are asked to scan the QR code before using the corresponding transportation tool or entering the corresponding place, so that the transmission chain can be easily recovered.

Specific comments –

Q3. Sample size –
The authors have not described how the sample size was estimated? Was the study adequately powered to predict the outcomes? It is recommended that a post-hoc power analysis be undertaken to assess if study is also adequately powered to meet the study objectives.

Reply: We have collected all 76936 confirmed cases in China including 3650 cases with known symptom onsets. The power of prediction of the proposed model has been verified in the manuscript. Besides, the prediction ability and comparison with simulation data are also given in the revised manuscript. As Q9 is about the simulation, please see the Reply to Q9 for details.

Q4. Sampling strategy –
How or on what criteria, the sample was selected should be described? Was it representative of the other COVID-19 patients in terms of profile and severity?

Reply: We collected all 76936 confirmed cases reported in official websites, which are the ensemble for the mainland China at that time. The official websites of provinces and cities are listed in Table R1. The 3650 confirmed cases with known symptom onsets are collected from the six provinces that have reported such information. Since all provinces except Hubei applied almost the same control measures, the samples are representative. In addition, this study is not closely related to the severity of patients and thus we have not consider such dimension.

Table R1. The official websites of provinces and cities.

Province	City	Website
Hebei	Wuhan	http://wjw.wuhan.gov.cn/front/web/list3rd/yes/802
	Xiaogan	http://wjw.xiaogan.gov.cn/xxgk/index.jhtml
	Huanggang	http://wjw.hg.gov.cn/col/col4668/index.html
	Jingzhou	http://www.jingzhou.gov.cn/zfwxw/jzyq/
	Suizhou	http://wjw.suizhou.gov.cn/news/list-4.html
	Xiangyang	http://wjw.xiangyang.gov.cn/zxzx/ztzl/xxfy/xxfb/

	Huanshi	http://wjw.huangshi.gov.cn/xwzx/wjyw/202002/t20200216_604360.html
	Jingmen	http://www.jingmen.gov.cn/col/col9452/index.html
	Yichang	http://wjw.yichang.gov.cn/list-41531-1.html
	Shiyan	http://wjw.shiyan.gov.cn/xwzx_13838/zhxx/
	Xiantao	http://wjw.xiantao.gov.cn/gsgg/
	Tianmen	http://www.tmwcn.com/html/news/tianmen/
	Enshi	http://wjw.enshi.gov.cn/zwjw/ywbb/
	Qianjiang	http://www.hbqj.gov.cn/wjwgsgg/
Guangdong	Shenzhen	http://wjw.sz.gov.cn/yqxx/
	Guanzhou	http://wjw.gz.gov.cn/ztzl/xxfyyqfk/yqtb/index.html
	Zhuhai	http://wsjkj.zhuhai.gov.cn/zwgk/tzgg/
	Dongguan	http://dghb.dg.gov.cn/gkmlpt/index#584
	Foshan	http://wjj.foshan.gov.cn/zwgk/gsgg/tzgg/
	Zhongshan	http://wjj.zs.gov.cn/xxml/ztzj/xxgzbdfk/fkdt/
	Huizhou	http://wjj.huizhou.gov.cn/zwgk/bmwj/tzgg/index.html
Henan	Xinyang	http://www.hnxywjw.gov.cn/index.html
	Nanyang	http://nyws.nanyang.gov.cn/info/iList.jsp?cat_id=43109
	Zhenzhou	http://wjw.zhengzhou.gov.cn/yqtb/index.jhtml
	Zhumadian	http://www.zmdwsj.gov.cn/pub/info/
	Shangqiu	http://www.shangqiu.gov.cn/Category_78/Index.aspx
	Zhoukou	http://www.hazhoukou.gov.cn/Home/gkml?code=gstg
	Pingdingshan	http://pdswsjsw.gov.cn/channels/10088.html
	Xinxiang	http://www.xxswjw.gov.cn/page206?article_category=5
	Anyang	http://aywjw.anyang.gov.cn/channels/71.html
Zhejiang	Wenzhou	http://wjw.wenzhou.gov.cn/col/col1209919/index.html
	Hangzhou	http://wsjkw.hangzhou.gov.cn/col/col1228996568/index.html
	Ningbo	http://wjw.ningbo.gov.cn/col/col142/index.html
	Taizhou	http://wsjkw.zjtz.gov.cn/gzdt/class/?68.html
	Jinhua	http://wjw.jinhua.gov.cn/xwzx/gzdt/
	Shaoxing	http://sxws.sx.gov.cn/col/col1228996673/index.html
	Jiaxing	http://wsjkw.jiaxing.gov.cn/col/col1228998388/index.html
Hunan	Changsha	http://wsjkw.changsha.gov.cn/ztzl_1/fkxxgzbd/index.html
	Yueyang	http://wsj.yueyang.gov.cn/11161/60061/index.htm
	Shaoyang	https://wjw.shaoyang.gov.cn/wjw/zyxw/list.shtml
	Changde	http://wjw.changde.gov.cn/zhdt/wjdt
	Zhuzhou	http://wjw.zhuzhou.gov.cn//c18748/index.html
	Loudi	http://wjw.hnloudi.gov.cn/ldjkh/xxgk/xxgk.shtml
	Yiyang	http://wjw.yiyang.gov.cn/13294/13300/default.htm
	Yongzhou	http://wjw.yzcity.gov.cn/
	Hengyang	http://wjw.hengyang.gov.cn/xxgk/gzdt/wjyw/
Anhui	Hefei	http://wjw.hefei.gov.cn/ztzl/xxgzbdgrdfyyqfk/xxfb/index.html
	Bengbu	http://wjw.bengbu.gov.cn/wsxx/
	Fuyang	http://wjw.fy.gov.cn/content/channel/5c35be0048787ad14d009195/

	Anqing	http://wjw.anqing.gov.cn/13971952.html
	Bozhou	http://wjw.bozhou.gov.cn/News/showList/5270/page_1.html
	Liuan	http://wjw.luan.gov.cn/zwzx/gsgg/index.html
Jiangxi	Nanchang	http://hc.nc.gov.cn/ncwjw/gzdt/nav_list.shtml
	Xinyu	http://wjw.xinyu.gov.cn/c103912/newlist.shtml
	Shangrao	http://www.srswjw.gov.cn/
	Jiujiang	http://wjw.jiujiang.gov.cn/zwzx_207/gggs/
	Yichun	http://wjw.yichun.gov.cn/
	Ganzhou	http://wjw.ganzhou.gov.cn/
	Fuzhou	http://wjw.jxfz.gov.cn/col/col4092/index.html?number=A00012
Jiangsu	Nanjing	http://rjg.njyh.gov.cn/14829/list.htm
	Suzhou	http://www.suzhou.gov.cn/szsrmzf/jbfk/nav_list.shtml
	Xuzhou	http://ws.xz.gov.cn/
	Huaian	http://wjw.huaian.gov.cn/wjyw/wjyw/list.html
	Wuxi	http://wjw.wuxi.gov.cn/zfxxgk/gggs/index.shtml
	Changzhou	http://www.changzhou.gov.cn/ns_class/jjfkxxfy2
	Lianyungang	http://www.lyg.gov.cn/lygswjw/
Chongqing	Wanzhou	http://wsjkw.cq.gov.cn/yqxxyqtb/
Shandong	Qingdao	http://wsjsw.qingdao.gov.cn/n28356065/n32563060/n32563061/index.html
	Jining	http://wjw.jining.gov.cn/col/col17066/index.html
	Linyi	http://wsjsw.linyi.gov.cn/gzdt/xwfb.htm
	Ji'nan	http://jnmhc.jinan.gov.cn/col/col50366/index.html?uid=18878&pageNum=1
	Yantai	http://wjw.yantai.gov.cn/col/col5653/index.html
	Weifang	http://www.weifang.gov.cn/ZT/RDZT/gzbdfyzt/tzgg/
Heilongjiang	Harbin	http://www.harbin.gov.cn/
	Shuangyashan	http://www.shuangyashan.gov.cn/
Beijing	Haidian	http://wjw.beijing.gov.cn/xwzx_20031/wnxw/
	Chaoyang	http://wjw.beijing.gov.cn/xwzx_20031/wnxw/
	Xicheng	http://wjw.beijing.gov.cn/xwzx_20031/wnxw/
Shanghai	Pudong	http://wsjkw.sh.gov.cn/xwfb/index.html
Hebei	Tangshan	http://m.ts.bendibao.com/news/xinxingfeiyan/
	Cangzhou	http://www.czscdc.com/list/?4_1.html
Fujian	Fuzhou	http://www.fuzhou.gov.cn/zgfzzt/swjw/fzwj/wjgg/
	Putian	http://wjw.putian.gov.cn/zwgk/qwbd/
	Quanzhou	http://health.quanzhou.gov.cn/xwzx/tzgg/
Guangxi	Nanning	http://wjw.nanning.gov.cn/gzdt/bjdt/index.html
	Beihai	http://xxgk.beihai.gov.cn/bhswshjhsywyh/tzgg_84785/
Shanxi	Xi'an	http://xawjw.xa.gov.cn/gzdt/wjyw/1.html
Yunnan	Kunming	http://wsjkw.km.gov.cn/
Hainan	Shanya	http://wst.hainan.gov.cn/yqfk/index/index/qianyi.html
Guizhou	Guiyang	http://www.gzhfpc.gov.cn/xwzx_500663/tzgg/index.html
Shanxi	Jinzhong	http://wjw.sxjz.gov.cn/gzdt/wjyw
Tianjin	Baodi	http://wsjk.tj.gov.cn/col/col87/index.html?uid=259&pageNum=1

Liaoning	Shenyang	http://wjw.shenyang.gov.cn/
Gansu	Lanzhou	http://wjw.lanzhou.gov.cn/col/col14897/index.html
Jinin	Changchun	http://wjw.changchun.gov.cn/

Q5. Methods –

The authors have not specified what type of distribution their data followed (normal/uniform/discrete/triangular/Beta-PERT distribution), this will determine how to output a random variable that follows a certain distribution. The authors should specify this and accordingly justify the method used. Did they use any of the data transformation methods? If so this should be specified

Reply: Based on the 3650 cases, the distribution of time intervals between symptom onsets and confirmations was empirically analyzed. The distribution can be well approximated by translational Weibull distribution

$$p(t_\Delta) = \frac{\alpha}{\beta}\left(\frac{t_\Delta + \gamma}{\beta}\right)^{\alpha-1} e^{-\left(\frac{t_\Delta+\gamma}{\beta}\right)^{\alpha}}, \qquad \text{(R1)}$$

where the shape parameter $\alpha \approx 1.48$, scale parameter $\beta \approx 7.03$ and translational parameter γ=0.10, as shown in Fig. R1. Note that, the distributions of time intervals may be different in different periods and for different countries. In addition, according to the empirical data reported by [Li Q, et al. Early transmission dynamics in Wuhan, China, of novel coronavirus infected pneumonia. N. Eng. J. Med. 2020; 382: 727–733.], the distribution of generation intervals follows a Gamma distribution

$$q(t_g) = \frac{\beta^{\alpha}}{\Gamma(\alpha)} t_g^{\alpha-1} e^{-\beta t_g} \quad (t_g > 0), \qquad \text{(R2)}$$

with the shape parameter $\alpha \approx 4.866$ and inverse scale parameter $\beta \approx 0.649$.

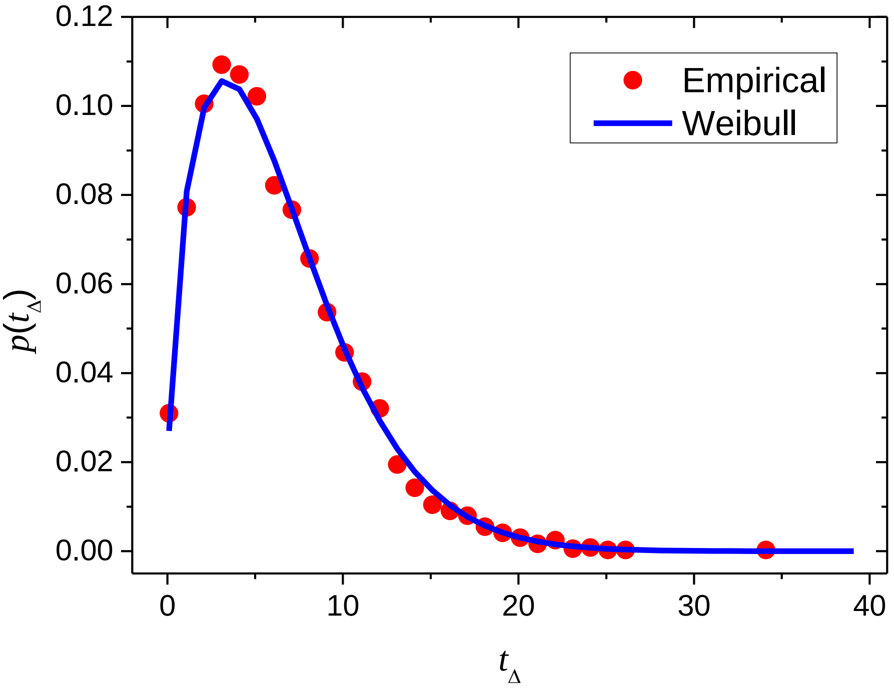

Fig. R1. Comparison between the empirical distribution of intervals between symptom onsets and confirmations (red circles) and the fitting curve (blue curve) that obeys the translational Weibull distribution.

Q6. Results –

• As Monte Carlo method is a probabilistic method with randomness playing a role in predicting future outcomes, there will always be a margin of error related to the results. The authors should specify the margin of error and confidence probability of valid findings.

Reply: According to your suggestion, we have analyzed the marginal error and confidence probability in the revised manuscript. Specifically, we take the interval time between the symptom onset and confirmation as the statistic variable **X**, and use K-S test [Crutcher H L. A note on the possible misuse of the Kolmogorov-Smirnov test. J Appl. Meteoro. 1975; 14: 1600-1602.] to estimate the marginal error ε , as

$$\varepsilon = D_\alpha \sigma \,, \tag{R3}$$

$$D_\alpha = 0.888/\sqrt{S} \,, \tag{R4}$$

$$\sigma = \sqrt{\frac{1}{S}\sum_{i=1}^{S} X_i^2 - \left(\frac{1}{S}\sum_{i=1}^{S} X_i\right)^2} \,, \tag{R5}$$

where S is the sample size, σ is the standard deviation, α is the significance level, and D_α is the critical value. In our work, the marginal error is ε=0.0379 subject to α=0.05 and S=10000.

• Q7. What was the accuracy of this proposed new method to the existing methods for simulation to calculate R0 that the authors have described.

Reply: Basic reproduction number R_0 is defined as the average number of secondary cases generated by an index case in a completely susceptible population without any interventions. It describes the spreading ability of the disease in the early stage of outbreak. Rather than R_0, this manuscript focuses on the effective reproduction number R_t, defined as the average number of secondary cases infected by an index case with symptom onset at time *t*. R_t is usually used to evaluate the efficacy of control measures.

In order to evaluate the performance of our method, we compare our method with the well-known Wallinga-Teunis method [Wallinga J, Teunis P. Different epidemic curves for severe acute respiratory syndrome reveal similar impacts of control measures. Am. J. Epidemiology. 2004; 160: 509–516]. Using simulation data as the benchmark, the comparison suggests that our method is more accurate than the Wallinga-Teunis method. For more details about the simulation model and results, please refer to the Reply to Q9.

•Q8. Kindly describe how exactly can/must we define the inputs and model the underlying processes to use this proposed new method?

Reply: According to this valuable suggestion, we have illustrated the inputs, output and processes of the proposed model in detail in the revised manuscript, so as to help readers understanding and repeating the method easily. In our model, the input includes the distribution of generation interval, the symptom onset times of some cases, and the confirmation times of all cases. The output of the model is the estimated effective reproduction number R_t. In the processing, we estimate the distribution of intervals between symptom onset times and confirmation times based on the samples with known symptom onsets and confirmation times (i.e., the above-mentioned 3650 cases) and apply the Monte Carlo sampling method to estimate the symptom onset times of other cases (i.e., the 76936-3650 cases without onset information) based on their confirmation times. So that, the epidemic curve of all cases can be approximately obtained. Finally, the effective reproduction number is estimated according to the epidemic curve and the distribution of generation intervals. The inputs, output and processing of the proposed method are illustrated in Fig R2.

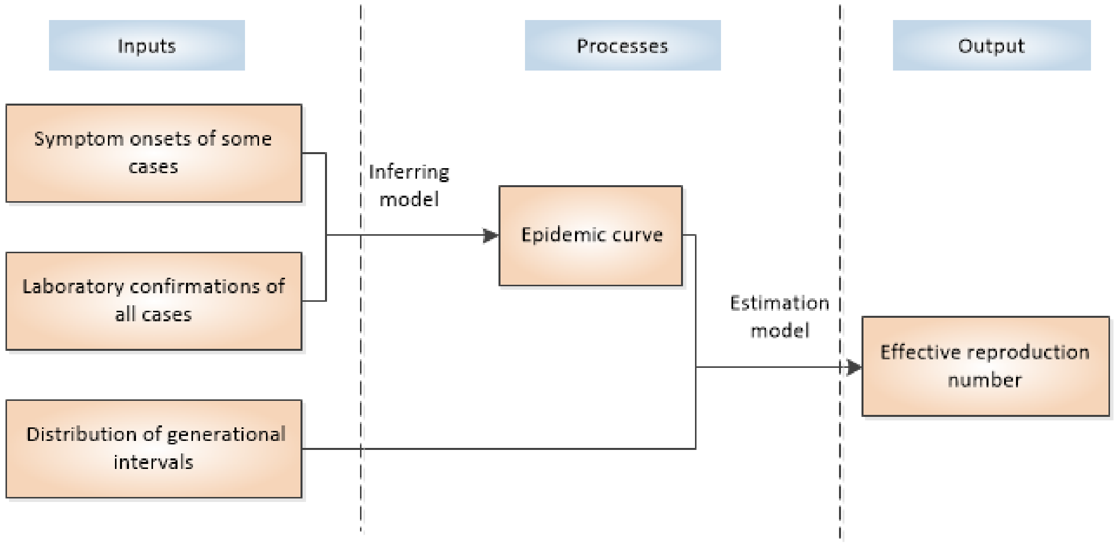

Fig. R2. The inputs, output and processes of the proposed method.

• Q9. It is recommended that tallying of Simulation results be done to establish reliability

Reply: Thank you very much for this constructive suggestion. We thus propose a so-called 5Γ-model with N=1,000,000 individuals to illustrate the reliability of the present method. The spreading starts with 10 initially infected individuals, and all infected and susceptible individuals are fully mixed. In the simulation, in each time step (i.e., a day), the number of contacted individuals of each infected case is independently drawn from the Gamma distribution Γ_1. For each contact between an infected individual and a susceptible individual, the infected probability is independently drawn from the Gamma distribution Γ_2. The time intervals between symptom onsets and laboratory confirmations obey the Gamma distribution Γ_3. The generation intervals obey the Gamma distribution Γ_4. The time intervals between laboratory confirmations and removals from the dynamics (i.e., died, recovered, effectively isolated, etc.) obey the Gamma distribution Γ_5. The means and variances of all the five Gamma distributions are listed in Table R2.

Table R2. The means and variances of the five Gamma distributions used in the simulation model.

Distribution	mean	variance
Γ_1	15	10
Γ_2	0.009	1.8×10^{-6}
Γ_3	5	2
Γ_4	7.5	3.4
Γ_5	20	8

The time window being analyzed is set to be [1, 40]. We assume that the symptom onsets of 20% randomly selected confirmed cases are known, and the confirmation times of all cases are known. The effective reproduction number R_t can be directly counted by the simulation model as all transmission chains are known. R_t can also be estimated by our method. Figure R3 compares the effective reproduction numbers directly counted based on the simulation and estimated by our method. One can see from Fig. R3 that our estimations agree very well with the benchmark. We have checked that our estimations work well subject to other reasonable chosen distributions and parameter settings.

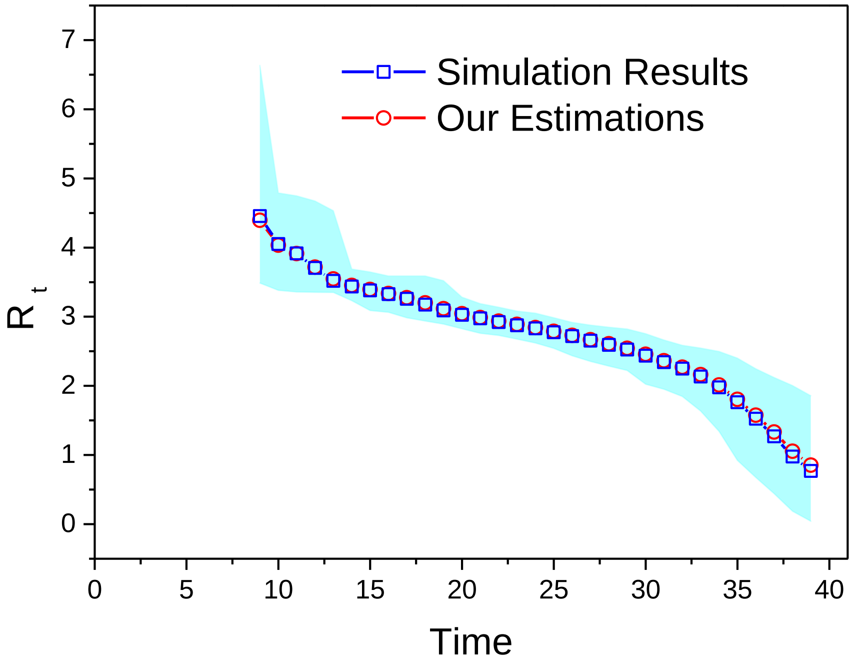

Fig. R3. The comparison of effective reproduction numbers directly counted based on the simulation results (blue squares) and estimated by our method (read circles). Our estimations are averaged over 10000 independent runs, and the cyan area denotes the 95% confidence intervals.

Q10. Discussion

• Is the Monte Carlo method that uses a stochastic model to your data? should be discussed

Reply: Yes, we use the uniform stochastic model U(0,1) to sample time intervals in our method. We first collect records with both symptom onsets and confirmed dates from the public reports, and then obtain the distribution $p(t_\Delta)$, where t_Δ denotes the time interval between symptom onsets and laboratory confirmations, which is taken as a non-negative integer for simplicity. According to the real data, the distribution $p(t_\Delta)$ can be approximated by a translational Weibull distribution. So, in the sample process, we use uniform stochastic model U(0,1) to return a random number z between 0 and 1, and then the time interval δt is defined by the

constrain $P(\delta t-1)<z\le P(\delta t)$,where $P(t_\Delta)$ is the cumulative distribution corresponding to $p(t_\Delta)$.

The rationality of such sample strategy is verified by comparing our results with the simulation model (see also the Reply to Q9). The cumulative distribution $p(t_\Delta)$ is obtained by randomly choosing 20% confirmed cases whose symptom onsets are known. The symptom onsets of the other 80% confirmed cases are estimated by the uniform stochastic model. It can be seen from Fig. R4 that the epidemic curve obtained by the uniform stochastic model agrees very well with the one by the simulation model. According to the reviewer and also for the convenience of readers, we add some introduction about stochastic sampling details in the revised version.

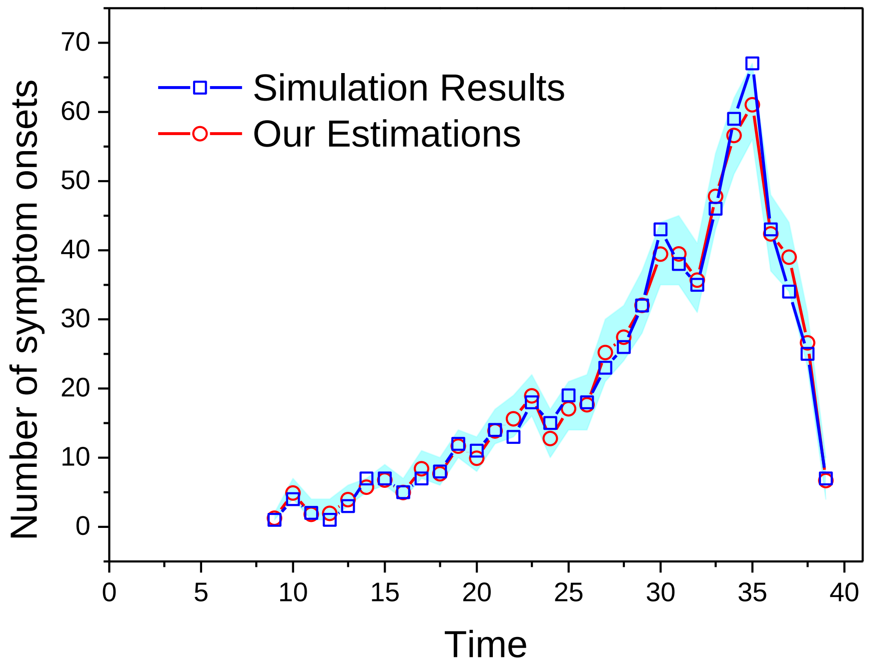

Fig. R4. The comparison of the epidemic curves (the number of cases with symptom onsets at each day) obtained by the simulation and our stochastic sampling method. The results are averaged over 10000 independent runs, and the cyan area denotes the 95% confidence intervals. The curves drop after *t*=35 because some cases are not yet confirmed before *t*=40 (our time window only covers [1,40]).

•Q11. Discuss the accuracy of your proposed method study vis-à-vis the accuracy of other established methods.

Reply: We compare the accuracy of our method and that of the Wallinga-Teunis method, with simulation results being the benchmark (see the Reply to Q9 for the simulation model). As shown in Fig. R5, the effective reproduction numbers estimated by our method are very close to the

benchmark values and remarkably more accurate than those obtained by the Wallinga-Teunis method.

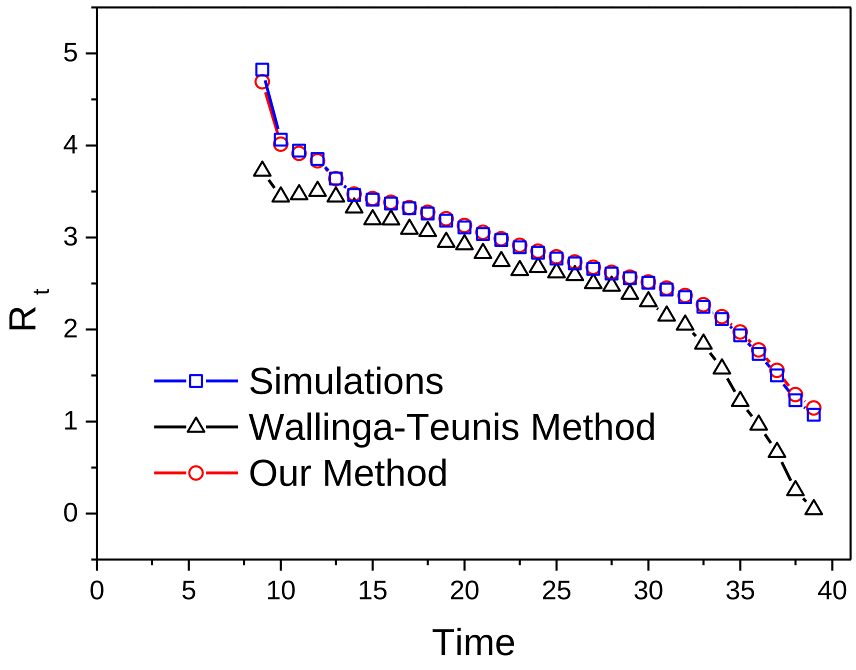

Fig. R5. The comparison of effective reproduction numbers directly counted based on the simulation results (blue squares) and estimated by the Wallinga-Teunis method (black triangles) and our method (red circles). The results obtained by the Wallinga-Teunis method and our method are both averaged over 10000 independent runs.

• Q12. Strengths and Limitations of the study should be discussed

Reply: We have added discussion about core strength and limitation in the revised version. *Strength*.—By introducing a Monte-Carlo method to estimate the symptom onsets of confirmed cases based on a small number of cases with known onsets, our method can utilize the information of all cases to calculate the effective reproduction number. In comparison, the Wallinga-Teunis method can only make use of the cases with both known symptom onsets and confirmation times. As shown in Fig. R5, our method produces obviously more accurate results than the Wallinga-Teunis method. *Limitation*.—One underlying assumption in our method is that the small number of samples are representative of all cases. This is a reasonable assumption for mainland China since control measures in different provinces are very much the same, all executing directives from the central government. However, in general, if the samples and the inferred cases are in different spreading stages or different areas, the reliability of the present method has to be carefully checked before any applications.

•Q13. Study is conducted in a small sub-set of Chinese population, limitations related to external generalizability should be discussed

Reply: The results are not sensitive to the ratio of samples to the total population, but the ratio of cases with known symptom onsets to all confirmed cases (4.74% in this study) and the absolute number of cases with known symptom onsets. As shown in the empirical analyese in the original submission, as well as the comparison with simulation results as suggested by the reviewer, those samples are enough to estimate the effective reproduction number if they are representative to (i.e., randomly sampled from) all confirmed cases. However, all the 3650 cases are obtained from 6 provinces, instead of a random sampling from all confirmed cases. However, as we have mentioned in the Reply to Q4 and Q12, it is reasonable to assume that those samples are representative since all provinces applied almost the same control measures. However, one should be aware of that this may be not the case of other country like US because different states may adopt different controlling strategies and launch different control measures. We have added some related discussion in the revised version.
