# Supplementary material for: Evaluating the effect of Chinese control measures on COVID-19 via temporal reproduction number estimation
Source: PLoS One. 2021 Feb 11;16(2):e0246715. doi: 10.1371/journal.pone.0246715 (PMC7877593; doi:10.1371/journal.pone.0246715)
Supplement: Attachment — Submitted filename: Reply-PONE-D-20-11733R1.pdf [file pone.0246715.s003.pdf]

Dear Editors,

Thank you very much for processing our revised manuscript entitled “Chinese Control Efficacy on COVID-19”. We have considered all the points and revised the manuscript accordingly. Enclosed please find a detailed response. For the sake of convenience, modifications are marked in red in the revised manuscript.

1. We note that the authors state in their abstract "The province-level analysis indicates that Chinese control measures on COVID-19 are very effective and efficient, that is, the effective reproduction numbers of the majority of provinces in mainland China got down to < 1 just by one week from the setting of control measures, and the temporal reproduction number of the week [15 Feb, 21 Feb] is only about 0.18" and also state in their discussion "The results indicate that Chinese control measures have achieved remarkable success..." and "The huge success of Chinese control measures on COVID-19 resulted from the ambitious and aggressive government-led actions." However, their study does not directly test whether specific control measures caused the reduction of R and new cases, and thus, we do not feel that these statements are supported by the rest of the study. To meet our publication criteria that conclusions are supported by the data (https://journals.plos.org/plosone/s/criteria-for-publication#loc-4) we recommend that authors change these sentences to something such as:

Abstract:
"The province-level analysis indicates that the effective reproduction numbers of the majority of provinces in mainland China got down to < 1 just by one week from the setting of control measures, and the temporal reproduction number of the week [15 Feb, 21 Feb] is only about 0.18. It is therefore likely that Chinese control measures on COVID-19 were effective and efficient, though more research needs to be performed."

Discussion:
"Our results suggest that Chinese control measures have been effective..." and
"Government-led actions likely played a role in the reduction of new COVID-19 cases."

Reply: Thank you very much for this suggestion, and we have revised corresponding statements in Abstract and Discussion to meet the publication criteria of PLoS ONE. In the abstract, the following statement "The province-level analysis indicates that Chinese control measures on COVID-19 are very effective and efficient, that is, the effective reproduction numbers of the majority of provinces in mainland China got down to < 1 just by one week from the setting of control measures, and the temporal reproduction number of the week [15 Feb, 21 Feb] is only about 0.18." is replaced by "The province-level analysis indicates that the effective reproduction numbers of the majority of provinces in mainland China got down to < 1 just by one week from the setting of control measures, and the temporal reproduction number of the week [15 Feb, 21 Feb] is only about 0.18. It is therefore likely that Chinese control measures on COVID-19 are effective and efficient, though more research needs to be performed."

In the section Discussion, we revise "The results indicate that Chinese control measures have

achieved remarkable success, with daily number of confirmed cases quickly decreasing after a short expansion lasting about two weeks from 21 January 2020." as "Our results suggest that Chinese control measures are likely to be effective and efficient, with daily number of confirmed cases quickly decreasing after a short expansion lasting about two weeks from 21 January 2020." Analogously, the statement "The huge success of Chinese control measures on COVID-19 resulted from the ambitious and aggressive government-led actions." is replaced by "Government-led actions likely played a role in the reduction of new COVID-19 cases.".

2. We also note that PLOS' guidelines state that the title should be "specific, descriptive, concise, and comprehensible to readers outside the field" (https://journals.plos.org/plosone/s/submission-guidelines#loc-title). In this case, we feel that the title is vague and does not describe the methods or aims of the study. We suggest that the title include a reference to the methodology or component to be measured (i.e., temporal reproduction number), the aim of the study (i.e, evaluating the effect of implementing COVID-19 control measures on reproduction number"), and the locale that was studied (i.e., China). For example, a title such as "COVID-19 control measure implementation in China: estimating the effect on temporal reproduction number" would be appropriate.

Reply: According to this suggestion, we use a more specific title as "Evaluating the effect of Chinese control measures on COVID-19 via temporal reproduction number estimation".
